# Transformed Hairy Roots of the actinorhizal shrub *Discaria trinervis*: a valuable tool for studying actinorhizal symbiosis in the context of intercellular infection

**DOI:** 10.1186/1753-6561-5-S7-P85

**Published:** 2011-09-13

**Authors:** Leandro Imanishi, Alice Vayssières, Claudine Franche, Didier Bogusz, Luis Wall, Sergio Svistoonoff

**Affiliations:** 1 Programa Interacciones Biológicas, Departamento de Ciencia y Tecnología, Universidad Nacional de Quilmes R. Sáenz Peña 352, B1876BXD Bernal, Argentina; 2 Groupe Rhizogenèse, Unité Mixte de Recherche Diversité Adaptation et Développement des Plantes (DIADE), Institut de Recherche pour le Développement (IRD), 911 avenue Agropolis, BP 5045, 34394 Montpellier Cedex 5, France

## Background

Nitrogen is a major limiting factor of plant growth in many ecosystems. Root nodule symbiosis (RNS) is one of the most efficient adaptations allowing plants to cope with nitrogen deficiency by establishing a symbiotic association with diazotrophic bacteria able to produce ammonium from atmospheric nitrogen. Nevertheless RNS is restricted to two groups of plants: legumes and Parasponia (*Celtidaceae*), that interact with a group of gram-negative proteobacteria collectively called rhizobia, and actinorhizal plants, a group of 220 species, mostly shrubs and trees distributed in the orders Fagales, Cucurbitales and Rosales, that interact with gram-positive actinomycetes of the genus *Frankia*[1]. All these plants belong to the Rosid I clade, suggesting a common origin for the ability to establish RNS [2].

In recent decades a strong research effort focused on model legumes lead to the identification of key molecular actors involved in nodulation, including the bacterial signalling molecules, the Nod factors and several genes involved in the symbiotic signalling pathways [3]. Much less is known in non model legumes and actinorhizal plants, particularly in species that are not infected like model legumes through root hairs but show more ancestral infection mechanisms like crack entry or intercellular infection. Yet important cues regarding the diversity and evolution of RNS are being found precisely in these more primitive non-model systems [4,5].

Among infection mechanisms leading to root nodule symbiosis, the intercellular infection pathway is probably the most ancestral but also one of the least characterized [6,7] . Intercellular infection has been described in *Discaria trinervis*, an actinorhizal shrub belonging to the Rosales order [8]. To decipher the molecular mechanisms underlying intercellular infection with *Frankia*, we set up an efficient genetic transformation protocol for *D. trinervis* based on *A. rhizogenes*.

## Methods

We analyzed the susceptibility of *D. trinervis* to two strains of *A. rhizogenes*: A4RS, and ARqua1; both strains contained a pHKN29 plasmid with a 35S::GFP fusion [9]. The classic *in-vitro* inoculation was compared to an *ex-vitro* method reported to be successful in several plant species [10]. The functionality of the symbiosis was tested on composite plants by performing nodulation tests and acetylene reduction assays. Using this technique, we introduced the promoter of *MtEnod11*, a nodulin gene from *M. truncatula* widely used as a marker for early infection-related symbiotic events in model legumes [11].

## Results

Transgenic roots showing strong levels of GFP were obtained for all treatments. The *ex-vitro* method using Arqua1 was the best compromise to obtain a good co-transformation efficiency while minimizing the impact on root system architecture. Co-transformed roots were specifically and efficiently nodulated with *Frankia* and the resulting nodules were undistinguishable from non-transgenic nodules in terms of developmental timing, anatomy, nitrogen fixation and feedback control by nitrogen. The expression of reporter genes such as *GUS* and *GFP* could be easily detected within transgenic *D. trinervis* root systems. The promoter of *MtEnod11* retained its symbiotic activation in transgenic *D. trinervis* nodules. Similar results were obtained in *C. glauca*[12].

## Conclusions

These findings open new avenues to study the genetic mechanisms of intercellular root invasion and single cell infection, allowing detailed characterization of genes involved in *D. trinervis* nodulation and a better understanding of the most ancestral infection pathways leading to RNS. In addition, because *D. trinervis* belongs to the Rosales order, evolutionary comparisons can be made with plants belonging to the same clade but unable to nodulate (most *Rosaceae*), or with Parasponia sp., the only non-legume able to enter RNS with rhizobia. The transformed roots in *D. trinervis* with appropriate reporter genes would be a powerful tool to explore signaling mechanisms in symbioses with this ancestral infection mode [13] .
